# Health status of older adults with Type 2 diabetes mellitus after aerobic or resistance training: A randomised trial

**DOI:** 10.1186/1477-7525-9-59

**Published:** 2011-08-02

**Authors:** Cindy Li Whye Ng, E Shyong Tai, Su-Yen Goh, Hwee-Lin Wee

**Affiliations:** 1 Department of Physiotherapy, Singapore General Hospital, Outram Road, Singapore; 2 Department of Endocrinology, Singapore General Hospital, Outram Road, Singapore; 3 Department of Rheumatology & Immunology, Singapore General Hospital, Outram Road, Singapore; 4 Department of Pharmacy, Faculty of Science, National University of Singapore, Singapore

**Keywords:** Diabetes mellitus, Exercise training, SF-36

## Abstract

**Background:**

A prior study showed positive effects of resistance training on health status in individuals with diabetes compared to aerobic or no exercise, the exercise regimens were either different in volume, duration or rate of progression. We aimed to compare the effects of progressive resistance training (PRT) or aerobic training (AT) of similar volume over an 8-week period on health status (measured using the Short-form 36 Questionnaire) in middle aged adults with type 2 diabetes mellitus (T2DM).

**Findings:**

Sixty subjects aged 58 (7) years were randomised to PRT (n = 30) or AT (n = 30). General health and vitality were significantly improved in both groups (mean (SD) change scores for PRT were 12.2(11.5) and 10.5(18.2), and for AT, 13.3(19.6) and 10.0(13.1), respectively) and exceeded the minimally important difference of 5 points. The PRT group also had improved physical function and mental health status (mean (SD) change scores: 9.0(22.6), p < 0.05 and 5.3(12.3), p < 0.05, respectively), which was not observed in the AT group. However, the between group differences were not statistically significant.

**Conclusions:**

Both exercise regimens have positive impact on health status that correlated well with clinical improvement in patients with T2DM. PRT may have some additional benefits as there were significant changes in more domains of the SF-36 than that observed for the AT group.

**Trial Registration:**

ClinicalTrials.gov NCT01000519

## Background

In Asia, more than 100 million people were living with T2DM in 2007 [1]. The prevalence in Singapore is 8.2% in adults aged 18 to 69 years and is expected to rise [2]. It is important to assess the impact of interventions that affect blood glucose control on health status besides clinical outcomes such as glycemic control [3]. Exercise is considered a critical part of therapeutic lifestyle intervention in the treatment of individuals with type 2 diabetes mellitus (T2DM) [4,5]. Exercise has been shown to improve quality of life in special populations [6,7]. In patients with T2DM, it is recommended that patients undertake both aerobic training and progressive resistance training [4]. We have recently shown that both types of training improve metabolic control to a similar degree [8]. In a recent study by Reid et al, it appeared that resistance training had more beneficial effects on physical health status than aerobic training [9]. However, the differences in the effects were not statistically significant [9]. Furthermore, they did not attempt to ensure similar volume or duration of exercise in all groups.

The aim of this study was to compare the effects of progressive resistance training (PRT) and aerobic training (AT), of similar volume and duration, on health status in middle-aged patients with T2DM.

## Hypothesis

Contrary to Reid et al.'s findings, we hypothesised that PRT and AT of similar volume would have similar effects on health status.

## Methods

We analysed data from 60 subjects with T2DM who participated in a randomised trial of PRT vs AT over an 8-week period [8]. The PRT group undertook nine resistive exercises (three sets of 10 repetitions) at 65% of their assessed one repetitive maximum while the AT group underwent 50 minutes of aerobic training with a target heart rate of 65% of their age-predicted maximum heart rate [8]. The calorie expenditure of both exercise programs was estimated to be 3.5 kcal/kg body weight. More details on the exercise regimes are provided (Additional file 1, Table S1). The main outcomes and the description of the exercise regimen have already been published [8]. In that study, we found that glycosylated haemoglobin (HbA1C) reduced by 0.4(0.6)% and 0.3(0.9)% in PRT and AT group respectively, but there was no significant difference between the groups (-0.1%, 95% CI -0.5 to 0.3) [8]. Systolic blood pressure as well as aerobic fitness in the form of peak oxygen consumption (VO_2_peak) favoured the AT group more by 9 mmHg (95% CI 2 to 16) and 5.2 ml/kg (95% CI 0.0 to 10.4) respectively [8]. The PRT group showed a greater reduction in waist circumference by 1.8 cm (95% CI 0.5 to 3.1) [8]. In this secondary analysis, we report on the impact of PRT and AT on health status as measured by the SF-36 questionnaire. All subjects gave written informed consent.

### SF-36 Health status

The self-administered SF-36 is a 36 item scale that measures eight aspects of functional health due to physical or emotional problems [10]. The eight subscales are summarised into the physical component summary score (PCS) and mental component summary score (MCS) using weights derived from factor analysis. In a multi-cultural population like Singapore, combining the scores of a QOL instrument administered in different languages will increase the power and representativeness of such studies [11]. The English (United Kingdom) and Chinese (Hong Kong) SF-36 versions were found to be equivalent in bilingual Singapore Chinese [12,13] and have demonstrated construct validity in the Asian population of Singapore [14]. Thus both versions were used in our study. The PCS and MCS based on the Singapore population norm for the 60 subjects who completed their exercise session were calculated using a published scoring algorithm in our local population [15], and standardised to a mean of 50 and standard deviation of 10.

### Statistical Analyses

Intention-to-treat analysis was undertaken. Baseline values of the scores for SF-36 were carried forward for the 11 participants who dropped out. Differences within groups before and after exercise were compared using paired T-test while differences between groups before and after exercise were compared using independent T-test, with statistical significance set at p < 0.05. To provide evidence of the construct validity of the SF-36 in this study sample, we reported the differences in SF-36 scores by known-groups.

## Results

The baseline demographics of the subjects are presented in Table 1. Both groups were not significantly different (p > 0.05).

**Table 1:** Baseline Characteristics.

Characteristics	All (n = 60)	Progressive Resistance Training (n = 30)	Aerobic Training (n = 30)	P-value for difference between groups
Age (years)	58 (7)	57 (7)	59 (7)	0.289
Gender (n, males)	19	11	8	NA
Ethnic (n, Chinese)	39	22	17	NA
Highest education level (n)				
Primary	17	8	9	NA
Secondary	31	15	16	
Tertiary	12	7	5	
Duration of diabetes (years)	12 (9)	11 (9)	12 (9)	0.710
Weight (kg)	69.9 (13.9)	69.5 (14.2)	70.3 (13.8)	0.821
BMI (kg/m^2^)	27.6 (4.9)	27.4 (4.7)	27.8 (5.2)	0.783
Waist circumference (cm)	91.3 (11.4)	90.8 (11.2)	91.9 (11.6)	0.724
Blood glucose (mmol/L)	9.9 (2.8)	10.4 (3.1)	9.5 (2.5)	0.233
HbA1C (%)	8.7 (1.2)	8.9 (1.5)	8.5 (0.9)	0.200
Body fat by skinfold (%)	34.6 (7.0)	33.9 (7.8)	35.3 (6.3)	0.451
Peak volume of oxygen consumed (ml/kg)	33.1 (16.5)	32.8 (17.8)	32.3 (15.5)	0.913

NA: not applicable; BMI: body mass index; HbA1C: glycosylated haemoglobin

### Health status (SF-36) (Table 2)

**Table 2:** Mean (SD) of groups, mean (SD) difference within groups, and mean (95% CI) difference between groups.

Outcome	Groups				Difference within groups		P value within groups		Difference between groups	P value
			
	Week 0		Week 8		Week 8 minus Week 0		Week 8 minus Week 0		Week 8 minus Week 0	Between groups
			
	PRT(n = 30)	AT(n = 30)	PRT(n = 30)	AT(n = 30)	PRT	AT	PRT	AT	PRT minus AT	
Physical functioning	66.6(24.9)	73.7(18.6)	75.7(19.9)	78.0(20.8)	9.0(22.6)	4.3(15.4)	0.037	0.134	4.7(-5.3 to 14.7)	0.354
Role-Physical	70.8 (40.5)	72.5 (38.5)	78.3 (35.8)	81.7 (33.4)	7.5 (27.2)	9.2 (25.0)	0.142	0.054	-1.7 (-15.2 to 11.8)	0.806
Bodily pain	72.9(21.7)	67.0(24.0)	76.7(20.4)	73.3(24.2)	3.9(20.4)	6.2(18.9)	0.307	0.082	-2.4(-12.5 to 7.8)	0.643
General Health	54.5(17.0)	52.7(19.1)	66.7(15.7)	66.0(20.8)	12.2(11.5)	13.3(19.6)	0.000	0.001	-1.1(-9.5 to 7.3)	0.792
Vitality	55.3(21.7)	57.7(16.6)	65.8(14.7)	67.7(15.0)	10.5(18.2)	10.0(13.1)	0.004	0.000	0.5(-7.7 to 8.7)	0.903
Social functioning	84.2(22.7)	83.8(16.8)	88.3(16.4)	87.5(13.9)	4.2(23.1)	3.8(11.9)	0.330	0.095	0.4(-9.1 to 10.0)	0.930
Role-Emotional	84.4 (30.0)	87.8 (27.0)	93.3 (18.4)	90.0 (25.0)	8.9 (24.7)	2.2 (23.0)	0.058	0.601	6.7 (-5.7 to 19.0)	0.284
Mental health	77.3 (14.3)	79.5 (13.3)	82.7 (12.9)	82.5 (11.9)	5.3 (12.3)	3.1 (10.2)	0.024	0.109	2.3 (-3.6 to 8.1)	0.439
Physical component summary score	49.6 (1.3)	49.5 (1.4)	49.3 (1.4)	49.4 (1.2)	-0.3 (1.1)	-0.2 (1.0)	0.184	0.380	-0.1 (-0.6 to 0.4)	0.730
Mental component summary score	50.5 (3.8)	51.0 (3.5)	52.3 (3.2)	52.3 (3.2)	1.9 (3.4)	1.3 (2.7)	0.006	0.013	0.5 (-1.1 to 2.1)	0.512

General health, vitality and MCS were significantly improved over time in both groups and reached statistical significance (p < 0.05). Physical functioning and mental health were significantly improved over time in the PRT group (mean (SD) change score: 9.0(22.6), p = 0.037 and 5.3(12.3), p = 0.024, respectively) but not in the AT group. These effects exceeded the minimally important difference of 5 points [16] (Table 2). In addition, the difference in the effects of PRT and AT on role-emotional was clinical significant (6.7, 95% CI 5.7-19.0). However, the difference in the effects between groups did not reach statistical significance.

At baseline, the MCS of study subjects were slightly above the population norm of 50 and both forms of exercise resulted in a significant increase in the MCS from baseline (PRT group, p = 0.006; AT group, p = 0.013).

Correlation between the PCS and MCS with the parameters that showed improvement in the published study [8] is presented (Additional file 2, Table S2). The correlation between the PCS and the change in HbA1C for the PRT group was positive (0.389, p = 0.037) while that for the AT group was negative (-0.490, p = 0.006). There was significant correlation between the MCS and body fat by skinfold measurement in the PRT group (0.628, p < 0.001) and change in HbA1C in the AT group (0.474, p = 0.008). Additional file 3, Table S3 presents the baseline norm-based scores for SF-36 for all subjects.

## Discussion

In this study comparing the impact of PRT and AT on health status in a multi-ethnic Asian population, the PRT group showed significant improvement in physical functioning, general health, vitality and mental health while the AT group demonstrated significant change in general health and vitality after an eight week supervised exercise program. Both groups also showed significant improvement in the mental component summary score. The better outcomes observed in the PRT group could be due to several possibilities: i) the novelty of resistance training, ii) the resistance training increasing subjects ability to perform activities of daily living, and iii) the perception of the exercises being less monotonous than being on a exercise machine for 20 minutes.

To the best of our knowledge, this is the first randomized trial investigating the effect of AT versus PRT on health status in an Asian population. The improvement observed in general health and vitality is in contrast to a study by Hill-Briggs et al. [17] in 149 African Americans that found that despite improvement in clinical outcomes in T2DM, there was no change in SF-36 domains. A possible explanation was that the effect of exercise on health status was short-term rather than long-term as Hill-Briggs et al. [17] studied her subjects before and after a 2-year period while we followed our patients over eight weeks. Another previous study also found that aerobic exercise training did not have any benefit on health status [18]. This study had a small sample size of nine subjects with T2DM and the aerobic sessions ranged from 20 to 45 minutes [18]. In the study by Reid et al, participants had a wider range in baseline HbA1C (6.6 to 9.9%) than our study and the exercise was also gradually increased in intensity and duration over the intervention period (15 minutes increased to 45 minutes for the aerobic group and two sets of up to eight repetitions increased to three sets of up to eight repetitions in the resistance exercise group). They observed a clinically significant improvement in the PCS favouring the resistance group compared to the aerobic group (mean difference of 2.7 points; p = 0.048) [9]. Although we did not observe an improvement in the PCS with either form of exercise in our study, we did find an improvement in the physical functioning domain of the SF-36 (a major component of PCS) in those randomized to PRT, which is in line with the observed benefits of resistance exercise in the PCS observed by Reid et al. It is possible that the larger sample size of at least 50 subjects in each group in the study by Reid et al and the longer intervention period of six months [9] allowed them to detect an effect on PCS that we did not observe.

An important strength of our study is that we made an attempt to match both exercise regimens as closely as possible for volume, frequency and rate of progression which previous studies did not control for. Another strength is that we have conducted a randomized trial design. We acknowledge that, the absence of a control group might limit our ability to assess the true effects of exercise on health status. However, we do not believe that this prevents us from comparing the benefits of AT vs PRT, which was the aim of our study. The small sample size may also have limited our ability to detect important difference in health status between the two types of exercise. Differences between the groups on role emotional exceeded a minimal important difference of 5 points [16] but did not reach statistical significance. In addition, we have used the original SF-36 rather than the SF-36 version 2 in our study as our institution held licence for the former but not the latter. The SF-36 version 2 was introduced to correct deficiencies identified in the original SF-36 and improve measurement properties to increase clarity and sensitivity (e.g. the response categories in mental health and vitality scales were reduced from six to five). Hence, we may have underestimated the effects of AT and PRT on health status. The sustained effect of exercise on health status over time and the combined effect of aerobic and resistance exercise on health status have also not been evaluated in our study and should be explored in future studies. Nevertheless, we believe that we have added new information to the sparse literature available on the impact of different forms of exercise on health status of Asian patients with T2DM.

## Conclusions

Both aerobic and progressive resistance training improved general health and vitality subscales in SF-36, as well as the mental component summary score. Although there was no significant difference between the groups, it did appear that progressive resistance training had more beneficial effects as there were significant changes in more domains of the SF-36 than that observed for the aerobic training group.

## List of abbreviations

(T2DM): Type 2 diabetes mellitus; (SF-36): Medical Outcome Trust Short-Form 36-item version; (PRT): Progressive resistance training; (AT): Aerobic training; (QOL): Quality of life; (DARE): Diabetes Aerobic and Resistance Exercise; (HbA1C): Glycosylated haemoglobin; (PCS): Physical component summary score; (MCS): Mental component summary score.

## Competing interests

The authors declare that they have no competing interests.

## Authors' contributions

LWCN participated in the data collection, interpretation of the study results and has written the first draft of the manuscript. EST contributed to the study design and the editing of the manuscript. S-YG contributed to the study design. H-LW contributed to the interpretation of the data and the editing of the manuscript. All the authors read and approved the final manuscript.

## Supplementary Material

Additional file 1 **Details of the aerobic exercise and progressive resistance exercise interventions**. A table describing the exercise protocols of the aerobic exercise and progressive resistance exercise interventions. Click here for file

Additional file 2 **Correlation (Significance) of SF-36**. A table showing the correlations between the PCS and MCS scores and the parameters that showed significant improvement post exercise interventions that was reported in the previously published article[8]. Click here for file

Additional file 3 **Baseline Short-Form 36 Questionnaires Norm-based scores, Mean (SD)**. A table with the baseline scores (mean and SD) of all the eight domains of the Short-Form 36 Questionnaire. Click here for file
