# Supplementary material for: Health status of older adults with Type 2 diabetes mellitus after aerobic or resistance training: A randomised trial
Source: Health Qual Life Outcomes. 2011 Aug 2;9:59. doi: 10.1186/1477-7525-9-59 (PMC3199739; doi:10.1186/1477-7525-9-59)
Supplement: Additional file 1 — Details of the aerobic exercise and progressive resistance exercise interventions. A table describing the exercise protocols of the aerobic exercise and progressive resistance exercise interventions. [file 1477-7525-9-59-S1.DOC]

**Additional File 1, Table S1:** Details of the aerobic exercise and progressive resistance exercise interventions

|  | Progressive resistance exercise | Aerobic exercise |
| --- | --- | --- |
| Intensity | 65 to 70% of 1 repetitive maximum | 65 to 70% maximum heart rate as determined by the modified Bruce protocol test |
| Duration | One set of 10 repetitions for each of the nine resistive exercises completed in a circuit  3 rounds of the circuit were completed in a maximum of 50 minutes | 50 minutes (10 minutes on upright or recumbent bicycle and 20 minutes each on the treadmill and elliptical cycle) |
| Type / Mode | Quadriceps (seated leg press machine & straight leg raises)  Hamstrings (hamstring curls machine)  Biceps, triceps, anterior and middle deltoids (using free weights)  Hip abductors and extensors (gluteal machine) | Treadmill, stationary upright bicycle, stationary recumbent bicycle, cross trainer (elliptical cycle) |
| Both Interventions | | |
| Before exercise | Heart rate, blood pressure and glucose level (pin prick) | |
| Warm up | 3 minutes of stretches of quadriceps, hamstrings, calf, biceps, triceps and back, with each muscle group stretched twice, holding each stretch for 15 seconds  10 minutes of unloaded cycling | |
| Program | 8-week group exercise program at Singapore General Hospital Physiotherapy Gym | |
| Frequency | 2-3 times a week, Mondays, Wednesdays & Fridays | |
