# Supplementary material for: Health status of older adults with Type 2 diabetes mellitus after aerobic or resistance training: A randomised trial
Source: Health Qual Life Outcomes. 2011 Aug 2;9:59. doi: 10.1186/1477-7525-9-59 (PMC3199739; doi:10.1186/1477-7525-9-59)
Supplement: Additional file 2 — Correlation (Significance) of SF-36. A table showing the correlations between the PCS and MCS scores and the parameters that showed significant improvement post exercise interventions that was reported in the previously published article[8]. [file 1477-7525-9-59-S2.DOC]

**Additional File 2, Table S2**: Correlation (Significance) of SF-36

|  | Progressive Resistance Training | | Aerobic Training | |
| --- | --- | --- | --- | --- |
|  | Physical component summary  n = 30 | Mental component summary  n = 30 | Physical component summary  n = 30 | Mental component summary  n = 30 |
| HbA1C (%) | 0.389  (0.037) | -0.105  (0.587) | -0.490  (0.006) | 0.474  (0.008) |
| Waist circumference (cm) | -0.049  (0.798) | -0.055  (0.775) | 0.085  (0.656) | 0.014  (0.941) |
| Body fat by skinfold (%) | -0.354  (0.055) | 0.628  (0.000) | -0.239  (0.203) | 0.235  (0.210) |
| Body fat by bioimpedance (%) | -0.076  (0.692) | 0.253  (0.178) | -0.137  (0.472) | 0.294  (0.115) |
| Systolic blood pressure, SBP (mmHg) | 0.351  (0.057) | -0.223  (0.236) | 0.321  (0.083) | -0.107  (0.573) |
|  |  |  |  |  |
| Peak volume of oxygen consumed, VO_2_ peak (ml/kg/min) | 0.269  (0.151) | 0.345  (0.062) | 0.032  (0.866) | -0.057  (0.767) |
