# Supplementary material for: Health status of older adults with Type 2 diabetes mellitus after aerobic or resistance training: A randomised trial
Source: Health Qual Life Outcomes. 2011 Aug 2;9:59. doi: 10.1186/1477-7525-9-59 (PMC3199739; doi:10.1186/1477-7525-9-59)
Supplement: Additional file 3 — Baseline Short-Form 36 Questionnaires Norm-based scores, Mean (SD). A table with the baseline scores (mean and SD) of all the eight domains of the Short-Form 36 Questionnaire. [file 1477-7525-9-59-S3.DOC]

**Additional File 3, Table S3**: Baseline Short-Form 36 Questionnaires Norm-based scores, Mean (SD)

|  | Overall (n=60) | PRT (n=30) | AT (n=30) |
| --- | --- | --- | --- |
| Physical Functioning | 70.2 (22.1) | 66.7 (24.9) | 73.7 (18.6) |
| Role-Physical | 71.7 (39.2) | 70.8 (40.5) | 72.5 (38.5) |
| Bodily Pain | 70.0 (22.9) | 72.9 (21.7) | 67.0 (24.0) |
| General Health | 53.6 (18.0) | 54.5 (17.0) | 52.7 (19.1) |
| Vitality | 56.5 (19.2) | 55.3 (21.7) | 57.7 (16.6) |
| Social Functioning | 84.0 (19.8) | 84.2 (22.7) | 83.8 (16.8) |
| Role-Emotional | 86.1 (28.3) | 84.4 (30.0) | 87.8 (27.0) |
| Mental Health | 78.4 (13.7) | 77.3 (14.3) | 79.5 (13.3) |
